# A Clinical Practice Update on the Latest AAOS/ADA Guideline (December 2012) on Prevention of Orthopaedic Implant Infection in Dental Patients

**Published:** 2013-03

**Authors:** Sh Hamedani

**Affiliations:** Private Practice, Shiraz, Iran

**Keywords:** Guideline, Prostheses and Implants, American Dental Association, Antibiotic Prophylaxis

## Abstract

The American Academy of Orthopaedic Surgeons (AAOS) and the American Dental Association (ADA), along with 10 other academic associations and societies recently (December 2012) published their mutual clinical practice guideline “**Prevention of Orthopaedic Implant Infection in Patients Undergoing Dental Procedures.**” This evidence-based guideline ,detailed in 325 pages, has three recommendations and substitutes the previous AAOS guideline. The new published clinical guideline is a protocol to prevent patients undertaking dental procedures from orthopaedic implant infection. The guideline is developed on the basis of a collaborative systematic review to provide practical advice for training clinicians, dentists and any qualified physicians who need to consider prevention of orthopaedic implant (prosthesis) infection in their patients. This systematic review found no explicit evidence of cause-and-effect relationship between dental procedures and periprosthetic joint infection (PJI).

This LTTE wishes to present a vivid summary of AAOS/ADA clinical practice guideline as a clinical update and an academic implementation to inform and assist Iranian competent clinicians and dentists in the course of their treatment decisions, to enrich the value and quality of health care on the latest international basis.

“Evidence Insufficient to Recommend Prophylactic Antibiotics for Dental Patients with Orthopaedic Implants.” was one of the smashing headlines of the dental updates in the winter 2013 [4-7].

The American Academy of Orthopaedic Surgeons (AAOS) and the American Dental Association (ADA), along with 10 other academic associations and societies recently (December 2012) published their mutual clinical practice guideline “**Prevention of Orthopaedic Implant Infection in Patients Undergoing Dental Procedures**” [1, 3]. 

This 325-page evidence-based guideline has three recommendations and substitutes the previous AAOS guideline. The new clinical practice guideline was established using the published AAOS CPG (Clinical Practice Guideline) development process and also considering all the standards recommended for systematic reviews and clinical practice guidelines. The full guideline presents a comprehensive systematic review of available evidence directing on the prevention of orthopaedic implant(OI) infection in patients receiving dental procedures [1]. 

Jevsevar (Chairman of AAOS group) and Abt (on behalf of ADA group) [3] published an editorial to this guideline and described how the recommendations have been evidence-based. They believe that antibiotic prophylaxis recommendations, in the 2009 AAOS information statement, can only be regarded as an educational aid and not as an official guideline [3]. 

## SYNOPSIS OF THE NEW GUIDELINE

The workgroup, initially developed three recommendations for antibiotic prophylaxis in dental patients with joint replacements. These recommendations shaped the basic foundation for systematic reviews of the literature regarding the dental procedures and periprosthetic joint infection (PJI). The workgroup also determined detailed criteria for quality appraisal of the published data and consequently avoiding any bias. To avoid bias, the AA-OS uses specific words for its recommendations and gives rationals for their usage. Due to the limitations in available evidence, the three recommendations presente-d in the new guideline are classified as limited, inconclusive and consensus with one recommendation for each grade of evidence. Higher grade recommendations are comparatively rare within published CPGs. The work team emphasized that they did not suppose this new guideline to be an impartial document. All three recommendations should be integrated into the decision-making process to improve patient care. The guideline accentuates on the collaboration between the physicians, dentists and patients to plan a treatment based on the evidence, clinical findings and patient preferences [1-3]. The following guideline is a summary of the AA-OS-ADA recommendations for prevention of OI infection in patients receiving procedural dental treatments. 

**Figure 1:**
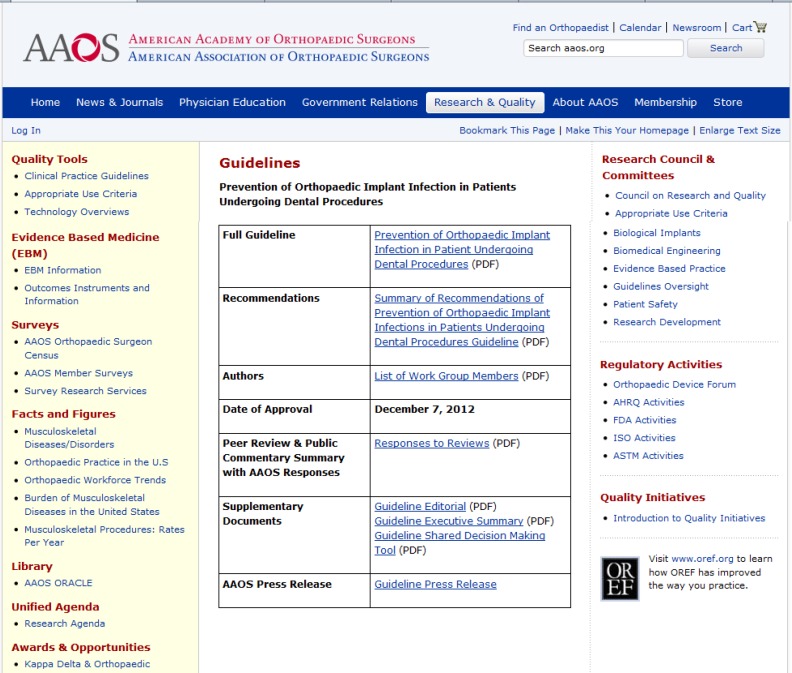
A print screen, copy image of the AAOS website to access the new guideline: http://www.aaos.org/research/guidelines/PUDP/ dental_guideline.asp

*Recommendation 1.*

**“The practitioner might consider discontinuing the practice of routinely prescribing prophylactic antibiotics for patients with hip and knee prosthetic joint implants undergoing dental procedures.” [1].**

Being graded as **Limited**, this recommendation is based on limited evidence and depicts that dental procedures are not related to OI infection. Moreover, it indicates that practitioners should consider changing their customary practice of prescribing prophylactic antibiotics for dental patients [1, 5-7]. The phrase limited is definitive; which means low levels of evidence is present to support the recommendation. Practitioners should be alert to up-coming publications that hold evidence and their decisions should reflect their individual judgment and the patient’s preferences [1].

Stronger evidences support this recommendation compared to other two recommendations:

I. Clinical practitioners believe in changing their longstanding tradition in the prescription of prophylactic antibiotics for dental patients.

II. The evidences indicate that dental procedures are not linked to the OI infections.

III. The risk of OI infections is not reduced by the pre-procedural antibiotic prophylaxis.

IV. Strong evidence indicates that pre-procedural antibiotic prophylaxis reduces the incidence of bacteremia induced by post dental procedure.

V. No evidence proves that bacteremia increases the risk of OI infections [1, 3, 5].

*Recommendation 2.*

**“**The work group was** unable to recommend for or against the use of topical oral antimicrobials in patients with prosthetic joint implants or other orthopaedic implants undergoing dental procedures.” [1]**

This recommendation is graded as Inconclusive, regarding the strength of the evidence. The guidelines implies that practitioners should consider a little constraint in their decision that whether to follow an **inconclusive** recommendation **or not.** The guideline emphasizes that patient preference should have a significant influencing role and practitioners should cautiously wait for future publications that elucidate the existing evidence to determine the balance between benefits and potential risk [1, 2, 4].

Apparently, this recommendation refers to the application of oral topical antimicrobials in the prevention of OI infections in dental patients. It indicates that there is no direct evidence to confirm that application of oral topical antiseptics (before dental procedures) would reduce bacteremia and hence prevent OI infections [5]. The guideline points out the followings as the examples of topical antiseptics administered by dentists: Chlorhexidine Gluconate oral rinse, povidone-iodine mouth rinse, hydrogen peroxide mouth rinse and mouth rinses with sodium-p-toluene (chloramine-T) [1-3, 5].

*Recommendation 3*

**“In the absence of reliable evidence linking poor oral health to prosthetic joint infection, it is the opinion of the work group that patients with prosthetic joint implants or other orthopaedic implants maintain appropriate oral hygiene.” [1]**

This recommendation was graded as **Consensus**, indicating that expert opinion supports the guideline recommendation albeit the fact that no available evidence can encounter the inclusion criteria. The guideline emphasizes on the imperative role of patients’ preference in decision making and also affirms the flexibility of practitioners in deciding whether to follow a recommendation rated as **Consensus **or not. Consensus recommendations are the weakest form of recommendation and cannot be used to ignore recommendations developed from higher levels of evidence [1-3]. 

This recommendation conveys the maintenance of good oral hygiene and apparently, it is the only consensus recommendation in the new guideline. Oral hygiene measures are available and cheap, provide possible benefit, are consistent with current clinical practice and are in concordance with good oral health [3].

**Goals and Implications for Clinical Practice**

The rate of OI infection is recorded from 0.3% to 8.3% in the available published literature. Invasion of organisms into the surgical wound during the surgery, haematogenous spread, recurrence of infection in previously involved and infected joints, or propagation from an infective local source may produce such infection [1].

Established on the best existing evidence, the rational for this clinical practice guideline is to assist the related clinicians and dentists to choose a paramount preventing and treatment modality when it is requisite. Contemporary dental practice inevitably depends on evidence-based standards and stipulates physicians and dentists to employ the best available evidence for treatment planning in their clinical practice. That’s why this guideline consists a systematic review of literature, conducted between October 2010 and July 2011 by AAOS and ADA methodologists and the doctor/dentist vocational groups and declared wherever the evidence was adequate or inadequate .They even discussed the gaps in the literature, where future researches are particularly needed [1].

Jevsevar, an orthopaedic surgeon and chairman of the AAOS team, declared that this clinical practice guideline was not supposed to be an impartial document and he confirmed that clinicians should use it as an instructive tool in their treatment planning to improve the quality and efficacy of their health care [3].

In summary, the guideline is deliberated to conduct clinical practice and also to provide a source of information for all qualified practitioners dealing with prevention of OI infection in dental patients. The AAOS and ADA hope that this guideline would also assist to ensure patients regarding the logics behind their treatment planning [1].

Therefore, The new guideline replaces the previous AAOS Information Statement and the full guideline with all succeeding credentials and workgroup declarations is available to access on the AAOS website: http://www.aaos.org/research/guidelines/PUDP/PUDP_guideline.pdf and the ADA website: http://www.ada. org/sections/professionalResources/pdfs/PUDP_guideline.pdf (Figure1).
